# Sparse Representation of Deformable 3D Organs with Spherical Harmonics and Structured Dictionary

**DOI:** 10.1155/2011/658930

**Published:** 2011-09-19

**Authors:** Dan Wang, Ahmed H. Tewfik, Yingchun Zhang, Yunhe Shen

**Affiliations:** ^1^Department of Electrical and Computer Engineering, University of Texas at Austin, Austin, TX 78712, USA; ^2^Department of Urologic Surgery, University of Minnesota, Minneapolis, MN 55455, USA

## Abstract

This paper proposed a novel algorithm to sparsely represent a deformable surface (SRDS) with low dimensionality based on spherical harmonic decomposition (SHD) and orthogonal subspace pursuit (OSP). The key idea in SRDS method is to identify the subspaces from a training data set in the transformed spherical harmonic domain and then cluster each deformation into the best-fit subspace for fast and accurate representation. This algorithm is also generalized into applications of organs with both interior and exterior surfaces. To test the feasibility, we first use the computer models to demonstrate that the proposed approach matches the accuracy of complex mathematical modeling techniques and then both ex vivo and in vivo experiments are conducted using 3D magnetic resonance imaging (MRI) scans for verification in practical settings. All results demonstrated that the proposed algorithm features sparse representation of deformable surfaces with low dimensionality and high accuracy. Specifically, the precision evaluated as maximum error distance between the reconstructed surface and the MRI ground truth is better than 3 mm in real MRI experiments.

## 1. Introduction

Organ deformation during operations has imposed substantial challenges for performing precise diagnosis and surgery in minimum invasive surgery (MIS), such as natural orifice transluminal endoscopic (NOTES) [1]. The deformation markedly decreases the precision of the prior surgical plan that is based on the preoperative images (e.g. computed tomography (CT) or magnetic resonance imaging (MRI)), so it must be effectively compensated to lower surgical risks. However, this is not a trivial task due to the high degree of freedom of the 3D nonrigid deformation and limited field of view [1] for observation during MIS. To recovery, the 3D deformation with high resolution in real time, a critical issue is to seek an efficient representation of deformable surface, according to which the sampling and surface recovery strategy can be designed for updating the 3D visualization. This paper focuses on the topic of block sparse representation of deformable surfaces. The later topic of real time tracking an deformable organ with limited access to the organ is explored further in [2–4]. 

 Various techniques have been proposed for surface description, and each has its own advantage and disadvantage according to the application requirements. Broadly speaking, there are two major categories of surface representation methods: local feature-based models and global or parametric models. The work in [5] based on geometric partial differential equations (PDE) belongs to the former category which derives Euler-Lagrange equation and then a geometric evolution equation (or geometric flow) to describe the surfaces. Similarly, the method in [6] treats the whole surface as a union of localized patches. Global surface representation methods [7–10], particularly, decomposing surfaces into other primitive shapes, are more appropriate for shape analysis and classification due to the lower dimensionality of the parameter space. This paper falls into the category of parametric global surface description.

Parametric surface representation describes a surface in a single functional form, such that the surface is fully characterized by a set of parameters in a particular domain. Surface harmonics such as spheroidal harmonics, cylindrical harmonics, and spherical harmonics (SH) [7] are widely used as building blocks for global surface description. Each harmonic does not bear localized features but contributes to the entire shape description. Among those different types of harmonics, a well-known approach is the spherical harmonic decomposition (SHD), which has advantages of smoothness and high accuracy [7, 11]. With proper parameterization [11, 12], any genus-0 surface (The genus of a connected surface is an integer representing the maximum number of cuttings along nonintersecting closed simple curves without rendering the resultant manifold disconnected. It is equal to the number of handles on it, so a sphere is genus 0 and a torus is genus 1.) can be analyzed in the harmonic domain with reduced data dimensions. SHD has been widely used in applications related to surface description, including static modeling of kidney [8] and brain [9, 10], as well as spatial-temporal modeling of left-ventricular with known motion period. In [13], hemisphere is also applied to open surfaces.

Sparse signal representation has steadily gained attention over the years in the signal processing community. The aim is to find a representation which is sparse, or compact, such that most of the energy of a signal can be captured with only a few nonzero coefficients in a given dictionary. The first widely applied methods to seek sparse representation are greedy approaches, including matching pursuit (MP) [14], orthogonal matching pursuit (OMP) [15], and orthogonal least square (OLS) [16]. Those methods iteratively first select the most correlated element from dictionary and then remove the contribution of that element with decorrelation, before finding the next atom. Iteration terminates when any stopping criteria is met. The second type is global optimization algorithms, such as Basis Pursuit (BP) [17], FOCUSS [18] and Iterative Thresholding [19]. Global Optimization, in the approximate sense, relaxes the sparseness constrain, and its sparsity is a side-effect of the optimization. For example, the basis pursuit (BP) method approximates the *l*_0_-norm sparsity constraint with an *l*_1_-norm criteria, which effectively converts the problem into a convex optimization one, solved globally with linear programming. The orthogonal subspace pursuit (OSP) method [20] used in our paper belongs to the greedy category, which does not require prior knowledge of the dimension of the subspaces and combines the learned subspaces to produce a data-driven dictionary with good sparseness and generalizability.

Besides sparse decomposition algorithms as mentioned above, an equally important issue for sparse representation is how to select a dictionary for an application. The two main groups for dictionary design methods are structured dictionaries built out of common bases, and trained dictionaries that are inferred from the training data. For the common bases, it is well known that the wavelet transform can be used to generate sparse multiscale representations of images, the short-time Fourier transform (STFT) generates sparse time-frequency representation of speech signals, and the DCT is another transform that has been used for compression in audio coding algorithms due to its good compaction property. For dictionary learning, the applicable approaches include maximum likelihood estimation (MLE) [21], method of optimal directions (MOD) [22], maximum a-posteriori (MAP) [23], and so forth. Those methods attempt to generalize the type of considered signal with the basis identified from a representative training data set. The proposed approach is based on trained dictionary, since, to the best of our knowledge, there is no common basis in which random surfaces can be sparsely represented.

Although sparse representation has been widely applied in the fields of signal compression, image denoising, blind source separation, and compressed sensing, there is still very limited application in 3D surfaces [24]. In fact, the main statistically motivated surface modeling methods are based on principle component analysis which is not sparse [25–29]. Those methods first compute the mean shape and then build the model by establishing legal variations learned from a set of training data for a given type of images, such as bone [25]. With PCA, the major variations of the shape populations are described by the first few basis vectors, such that any surface of that shape population can be projected into an orthogonal subspace spanned by the retained vectors. More advanced techniques, such as multiresolution deformable model [27], are provided to improve the accuracy considering limited sample size.

 Most of the previous surface modeling/representation works are designed for either static models [8–10], or particular deformable organs with known physical properties (such as motion cycle) [30]. Further, computation bottleneck caused by large spherical harmonics basis hampered the applicability for real-time application. For PCA-based modeling methods, the resulting space that captures the variation in the population is either a super subspace including all training data or a truncated subspace with sacrificed generalization. In addition, PCA tends to be computational expensive when performing eigenvector decomposition as training data dimension increases, and it does not lead to any structure in the representation.

 To bring the demonstrated merits of sparse coding to 3D surface representation, we propose a generally applicable algorithm of parametric sparse representation of deformable surfaces based on SHD and orthogonal subspace pursuit. The main contributions of this paper include the following.

- Propose an algorithm of sparse representation of deformable surfaces.

(ii) Generalize the representation approach for organs involving both interior and exterior surfaces.

(iii) Present evaluation results conducted using computer models, ex vivo experiments based on 3D MRI scans of freshly excised porcine kidneys, and in vivo cardiac MRI scans of real patients.

This paper is organized as follows: in Section 2, we describe the proposed algorithm of sparse representation of deformable surface, denoted as SRDS thenceforth.  Section 3 presents some experimental results using finite element model (FEM) data, ex vivo and in vivo experimental data. Finally, in Section 4, we finish with a few conclusions.

## 2. The SRDS Algorithm

**Figure 1:**
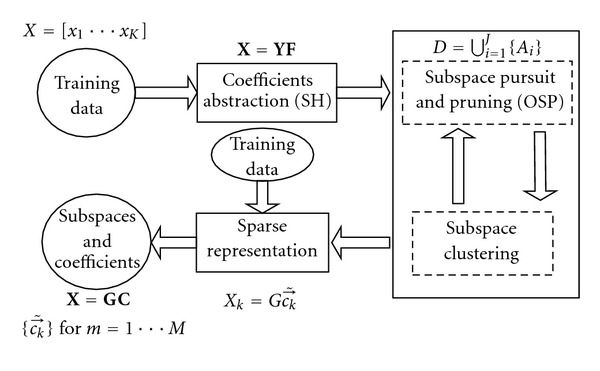
Flowchart of SRDS algorithm consists of three steps specified in the solid rectangulars; ellipsoids denote data input and output.

The SRDS algorithm consists of three main steps to achieve sparse representations of deformable surfaces, as outlined in Figure 1. Initially, SHD is performed to depict the deformable surfaces in the training set in the harmonic domain. Then OSP is applied in the transformed domain to identify the subspaces in which the SH coefficient vectors of the deformations can be linearly represented. Finally, each deformation is clustered to the proper subspace and represented with the corresponding coefficient vector with block sparsity. This representation method is also extended to organ deformations occurred on both interior and exterior layers as described in Section 2.4. Furthermore, as a practical issue, pixel-wise surface alignment among all the 3D surfaces is also addressed in Section 2.5.

### 2.1. Step 1: SHD

Spherical harmonics are solutions to Laplace's equation expressed in the spherical coordinate system, defined as

(1) $$\begin{array}{cc} Y_{lm}\left(\theta ,\varphi \right) & ={\left(-1\right)}^{m}\sqrt{\frac{2l+1}{4\pi }}\sqrt{\frac{\left(l-m\right)!}{\left(l+m\right)!}}P_{lm}\left(\cos\;\theta \right)e^{im\varphi }, \end{array}$$

where *θ* is the polar angle within [0, *π*], *φ* is the azimuthal angel within [0, 2*π*), *l* is the harmonic degree within [0, +*∞*], and *m* is the harmonic order varying in [−*l*, *l*]. *P*_*lm*_ is the associated Legendre function. After proper parameterization [11, 12], a 3D surface **x** with finite energy can be expanded with SH series as

(2) $$\begin{array}{cc} x\left(\theta ,\varphi \right) & =\overset{\infty }{\underset{l=0}{\sum }}\overset{+l}{\underset{\;m=-l}{\sum }}f_{lm}Y_{lm}\left(\theta ,\varphi \right). \end{array}$$

Each harmonic coefficient *f*_*lm*_ is calculated using the inner product of the function **x**(*θ*, *φ*) and basis *Y*_*lm*_(*θ*, *φ*)

(3) $$\begin{array}{cc} f_{lm} & =\overset{2\pi }{\underset{\varphi =0}{\int }}\overset{\pi }{\underset{\theta =0}{\int }}x\left(\theta ,\varphi \right)Y_{lm}\left(\theta ,\varphi \right)sin\theta \;d\varphi \;d\theta . \end{array}$$

Assume that harmonics up to level *L* (*l* ≤ *L*) are involved in the transformation. Let **Y** denote the matrix composed of all (*L*+1)^2^ discretized harmonics, so **Y** has the following formation:

(4) $$\begin{array}{cc} Y & ={\left|\begin{array}{cccccc} {\vec{Y}}_{0,0} & {\vec{Y}}_{1,-1} & {\vec{Y}}_{1,0} & {\vec{Y}}_{1,1} & \cdots & {\vec{Y}}_{L,L} \end{array}\right|}_{N\times {\left(L+1\right)}^{2}}. \end{array}$$

Then a surface can be represented in the matrix format as

(5) $$\begin{array}{c} x=Y\vec{f}, \end{array}$$

where **x** stands for a surface with *N* samples and $\begin{array}{cc} \vec{f} & ={\left[f_{0,0}\;f_{1,-1}\;f_{1,0}\;f_{1,1}\;\cdots \;f_{L,L}\right]}^{T} \end{array}$ is the harmonic coefficient vector. Notice that this equation is not exactly equal but approximately. For simplicity, we still use equal sign with least square estimation in this paper. The linear problem in (5) can be solved with the least square (LS) constraints outputting $\vec{f}$

(6) $$\begin{array}{c} \vec{f}={\left(Y^{T}Y\right)}^{-1}Y^{T}x. \end{array}$$

Perform SHD for each of the *K* training deformed surfaces in **X** = {**x**_*k*_}_*k*=1_^*K*^, so the group of deformations can be described by matrix **F**

(7) $$\begin{array}{c} F={\left|\begin{array}{cccc} {\vec{f}}_{1} & {\vec{f}}_{2} & \cdots & {\vec{f}}_{K} \end{array}\right|}_{{\left(L+1\right)}^{2}\times K}. \end{array}$$

Consequently, the training set of deformations can be totally characterized by columns in **F** as

(8) $$\begin{array}{cc} X & =YF. \end{array}$$

### 2.2. Step 2: Subspace Identification with OSP

The aim of the second step is to explore the structures in those training deformations in the transformed harmonic domain and recognize the inherent subspaces in which the SHD coefficient vectors of the training deformations can be projected with high accuracy. The newly developed OSP algorithm [20] is adopted since it features better generalization and less computational cost compared to the gold standard K-SVD algorithm [31]. OSP is an iterative process that terminates when one of the predefined criteria is met. In this paper, we specify the following two stopping criteria: (1) an error threshold for *ε* subspaces selection and (2) a maximum number of iterations *E*_max_ for both controlling the subspace dimensions and avoiding deadlock searching. Further, the threshold for vector clustering is denoted as *η*, that is, we declare that a vector lives in a subspace if it can be projected to that subspace with error (*l*_2_ distance) no larger than *η*.

**A. Subspace Pursuit**

Initially, each vector ${\vec{f}}_{k}$ of length (*L*+1)^2^ in **F** is normalized by *l*_2_ norm. For convenience, we still use **F** to denote SH coefficient matrix even after normalization. The algorithm first identifies a subspace from **F** based on the stop criteria, then finds all the vectors in **F** that can be represented in that subspace with error level below *η* and remove those vectors from **F** to prepare for the next subspace pursuit. The process can be generalized as follows, in which *A* ⊗ *B* means that elements from *B* are excluded from *A*, and *A* ⊕ *B* stands for inclusion.

(1) Initialization: *i* = 0, **D** = *∅*, **F**^0^ = **F**.
(2) Subspace searching and clustering
  - *i* = *i* + 1; choose a vector ${\vec{f}}_{i}$ from **F**^*i*^ (e.g. first column of **F**^*i*^) and let $F^{i}=F^{i-1}⊗{\vec{f}}_{i}$ to remove ${\vec{f}}_{i}$.
  - Find *n*_*i*_ vectors from $F^{\prime }=F⊗{\vec{f}}_{i}$ for representing ${\vec{f}}_{i}$ with error no larger than *ɛ* within *E*_max_ iterations and the *n*_*i*_ vectors form *S*_*i*_.
  - Perform SVD decomposition on *S*_*i*_: *U*Σ*V*^*T*^ = *S*_*i*_; let **A**_*i*_ contain the first *n*_*i*_ vectors of *U*; update **D** = **D** ⊕ **A**_*i*_.
  - Select vectors from **F**^*i*^ that can be represented by **A**_*i*_ with error no larger than *η*, and then remove them from **F**^*i*^.
  - Repeat above steps until all the vectors are clustered.

**B. Subspace Pruning**

One disadvantage of the traditional OSP algorithm is the presence of “spurious” or redundant subspaces especially as the dimension of the training data set increases. Those subspaces identified in the earlier iterations actually can be better represented by the later identified subspaces. Therefore, a postprocessing step is used to identify and then discard the redundancy among the subspaces without decreasing the performance. This is implemented by repartitioning the training data among the initial subspaces and then eliminating subspaces in which very few or no vector is clustered. In some cases, where the subspace size is limited to some constraint, an optimization step can be applied in conjunction with the pruning step. The details of the subspace optimization design is described in [20].

**C. Matrix F Factorization**

After identifying the inherent subspaces, the coefficient matrix **F** of training set can be partitioned into two-part union of subspaces and corresponding coefficients via the following procedures.

Since each vector ${\vec{f}}_{k}$ has been clustered into the belonging subspace during the subspace identification process, the corresponding coefficients for each ${\vec{f}}_{k}$ can be obtained accordingly. Suppose that ${\vec{f}}_{k}$ lives in subspace **A**_*i*_ which is spanned by *n*_*i*_ orthogonal basis, so its corresponding coefficients ${\vec{c}}_{k}$ can be calculated using LS estimator

(9) $$\begin{array}{c} {\vec{c}}_{k}={\left(A_{i}^{T}A_{i}\right)}^{-1}A_{i}^{T}{\vec{f}}_{k}. \end{array}$$

Then ${\vec{f}}_{k}$ can be characterized by ${\vec{c}}_{k}$ in its subspace

(10) $$\begin{array}{c} {\vec{f}}_{k}=A_{i}{\vec{c}}_{k}. \end{array}$$

**Figure 2:**
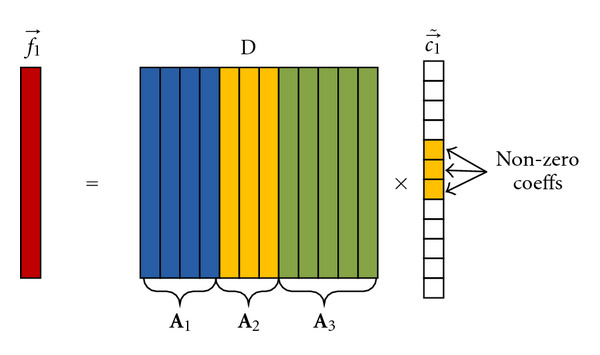
Sparsity of coefficient vector ${\tilde{\vec{c}}}_{1}$.

If there are totally *J* subspaces identified from **F**, a structured dictionary constructed by concatenating all deformation subspaces is established as **D** = ⋃_*i*=1_^*J*^{**A**_*i*_}, with dimension *I* = ∑_*i*=1_^*J*^*n*_*i*_. Since each vector ${\vec{f}}_{k}$ lies in one of the subspaces, ${\vec{f}}_{k}$ can also be represented in the structured dictionary with a block sparse vector $\left\{{\tilde{\vec{c}}}_{k}\right\}$, which is obtained via extending the coefficients ${\left\{{\vec{c}}_{k}\right\}}_{k=1}^{K}$ by zero padding in positions corresponding to other subspaces in **D**.  Figure 2 provides an example of 3 subspaces to illustrate the sparsity of coefficient vector ${\tilde{\vec{c}}}_{1}$. If ${\vec{f}}_{1}$ lies in subspace **A**_2_ which are spanned by the 5th, 6^th^, and 7th columns in **D**, then ${\tilde{\vec{c}}}_{1}$ has nonzero values only at index of 5, 6, and 7. Consequently, **F** can be factorized as

(11) $$\begin{array}{cc} F & =DC, \end{array}$$

where $\begin{array}{cc} C & ={\left|{\tilde{\vec{c}}}_{1}\;{\tilde{\vec{c}}}_{2}\;\cdots \;{\tilde{\vec{c}}}_{K}\right|}_{I\times K} \end{array}$ is the corresponding coefficient matrix with block sparsity.

### 2.3. Step 3: Structured Sparse Surface Representation

**A. Sparse Representation of Training Surfaces**

Integrating the subspace pursuing results in the harmonic domain in (11) with the initial SHD process in (8), the training deformations **X** can be sparsely represented in the original spatial domain as

(12) $$\begin{array}{cc} X & =YDC\\ & =GC, \end{array}$$

where **G** = **YD** with size of *N* × *I* is the desired structured dictionary in the spatial domain. Since **D** = ⋃_*i*=1_^*J*^{**A**_*i*_}, **G** is inherently structured by subspaces of **G** = ⋃_*i*=1_^*J*^{**G**_*i*_} with **G**_*i*_ = **YA**_*i*_ of size *N* × *n*_*i*_.

Up to this point, with matrix **G** that captures the deformation features in the considered population, each training deformation **x**_*k*_ in **X** can be fully characterized by a sparse coefficient ${\tilde{\vec{c}}}_{k}$ as

(13) $$\begin{array}{c} x_{k}=G{\tilde{\vec{c}}}_{k}. \end{array}$$

The sparsity of ${\tilde{\vec{c}}}_{k}$ has already been illustrated in Figure 2.

**B. Sparse Representation of Testing Surfaces**

For the testing deformations beyond the training set, we utilized the fact that the dictionary identified from an extensive training data features good generalization such that any deformation of that particular population can be represented in the subspaces with high accuracy. This is justified because organs only deform in limited ways due to their mechanical properties, so the deformation variations can be fully learned from a training data set. This applied structure allows fast deformation representation in subspaces of low dimensionality.

The testing set is denoted as **H** = {**h**_*m*_}_*m*=1_^*M*^, where *M* is the number of deformations to be represented. The straightforward strategy is to find a best-fit subspace for **h**_*m*_ by projecting it to every subspace {**G**_*i*_}_*i*=1_^*J*^ and choose the subspace with minimal projection error. Since the number of subspaces *J* and the dimension of each subspace {*n*_*i*_}_*i*=1_^*J*^ are both small with the postprocessing of subspace pruning, this best-fit strategy still results in low computational cost. However, when the number of subspaces is too large, an alternative threshold approach can be applied by finding a subspace **G**_*i*_ in which **h**_*m*_ can be represented with an error level smaller than *η*. The former best-fit method is implemented in Section 3 for performance validation.

Suppose that **G**_i*_ is the chosen subspace, so coefficient vector ${\vec{c}}_{m}$ can be estimated with LS as

(14) $$\begin{array}{c} {\vec{c}}_{m}={\left(G_{i^{*}}^{T}G_{i^{*}}\right)}^{-1}G_{i^{*}}^{T}h_{m}. \end{array}$$

Then block sparse vector

(15) $$\begin{array}{cc} {\tilde{\vec{c}}}_{m} & =\left[0\;0\;\cdots \;{\vec{c}}_{m}\;\cdots \;0\;0\;0\right] \end{array}$$

is obtained according to the rules described in Section 2.2. Further, the sparsity of ${\tilde{\vec{c}}}_{k}$ or ${\tilde{\vec{c}}}_{m}$ can be increased by trimming off nonzero elements with absolute value lower than a given threshold *δ*.

It is worth noting that, different from the traditional learning approaches relying on orthogonal least square (OLS) [32] or matching pursuit (MP) [33] algorithms which select atoms from the training set and recombine them for representing each surface in the testing set sparsely, our SRDS algorithm avoids this heavy overload caused by reshuffling all the atoms. Instead, we apply the block structure of the dictionary learned from a representative training data set. This essentially enables the representation of each deformable surface compactly and sparsely with high accuracy and low computational cost.

### 2.4. Extended Sparse Surface Representation

For an organ with both interior and exterior surfaces, such as bladder, deformations can take place on both layers. The above theory can be extended to achieve sparse surface representation for deformations occurred on both interior and exterior wall of the object.

Initially, spherical parameterization is conducted on interior and exterior parts separately. We denote ${\vec{x}}_{k}^{\text{in}}$ and ${\vec{x}}_{k}^{\text{ex}}$(1 ≤ *k* ≤ *K*) as the corresponding interior and exterior of each training surface ${\vec{x}}_{k}$. Then each pair of ${\vec{x}}_{k}^{\text{in}}$ (with *N*_1_ vertices) and ${\vec{x}}_{k}^{\text{ex}}$ (with *N*_2_ vertices) can be approximated by spherical harmonic basis as

(16) $$\begin{array}{c} {\vec{x}}_{k}=\left[\begin{array}{c} \hat{{\vec{x}}_{k}^{\text{in}}}\\ \hat{{\vec{x}}_{k}^{\text{ex}}} \end{array}\right]=\left[\begin{array}{cc} Y^{\text{in}} & O\\ O & Y^{\text{ex}} \end{array}\right]\cdot \left[\begin{array}{c} {\vec{f}}_{k}^{\text{in}}\\ {\vec{f}}_{k}^{\text{ex}} \end{array}\right] \end{array}$$

where **Y**^in^ of size *N*_1_ × (*L*+1)^2^ and **Y**^ex^ of size *N*_2_ × (*L*+1)^2^ denote the spherical harmonic basis for inner and outer surfaces, respectively. *L* is the highest degree of harmonics included. ${\vec{f}}_{k}^{\text{in}}$ and ${\vec{f}}_{k}^{\text{ex}}$ are the corresponding harmonic coefficient vectors. Therefore, each deformation is represented by vector $\begin{array}{cc} {\vec{f}}_{k} & =\left[\begin{array}{c} {\vec{f}}_{k}^{\text{in}}\\ {\vec{f}}_{k}^{\text{ex}} \end{array}\right], \end{array}$ and all *K* training frames can be characterized by $F=⋃{\left\{{\vec{f}}_{k}\right\}}_{k=1}^{K}$ as

(17) $$\begin{array}{c} X=YF=\left[\begin{array}{cc} Y^{\text{in}} & O\\ O & Y^{\text{ex}} \end{array}\right]\left[\begin{array}{c} {\vec{f}}_{1}^{\text{in}}\cdots {\vec{f}}_{K}^{\text{in}}\\ {\vec{f}}_{1}^{\text{ex}}\cdots {\vec{f}}_{K}^{\text{ex}} \end{array}\right]. \end{array}$$

The following procedures of subspace identification and sparse surface representation as described in Sections 2.2 and 2.3 can be applied straightforwardly. After identifying *J* subspaces **D** = ⋃_*i*=1_^*J*^{**A**_*i*_} from SH coefficient matrix **F**, each training deformation can be sparsely represented with block sparse coefficient vector ${\tilde{\vec{c}}}_{k}$ as:

(18) $$\begin{array}{cc} {\vec{x}}_{k}=\left[\begin{array}{c} \hat{{\vec{x}}_{k}^{\text{in}}}\\ \hat{{\vec{x}}_{k}^{\text{ex}}} \end{array}\right] & =\left[\begin{array}{cc} Y^{\text{in}} & O\\ O & Y^{\text{ex}} \end{array}\right]D{\tilde{\vec{c}}}_{k}\\ & =G{\tilde{\vec{c}}}_{k}=G_{i}{\vec{c}}_{k}, \end{array}$$

where $\begin{array}{cc} G_{i} & =\left[\begin{array}{cc} Y^{\text{in}} & O\\ O & Y^{\text{ex}} \end{array}\right]A_{i} \end{array}$ is the subspace with size of (*N*_1_ + *N*_2_) × *n*_*i*_, and **G** = ⋃_*i*=1_^*J*^{**G**_*i*_} is the desired structured dictionary. Accordingly, ${\tilde{\vec{c}}}_{k}$ is the block sparse vector, and ${\vec{c}}_{k}$ is the nonzero coefficient values in the selected subspace.

### 2.5. Surface Correspondence

**Figure 3:**
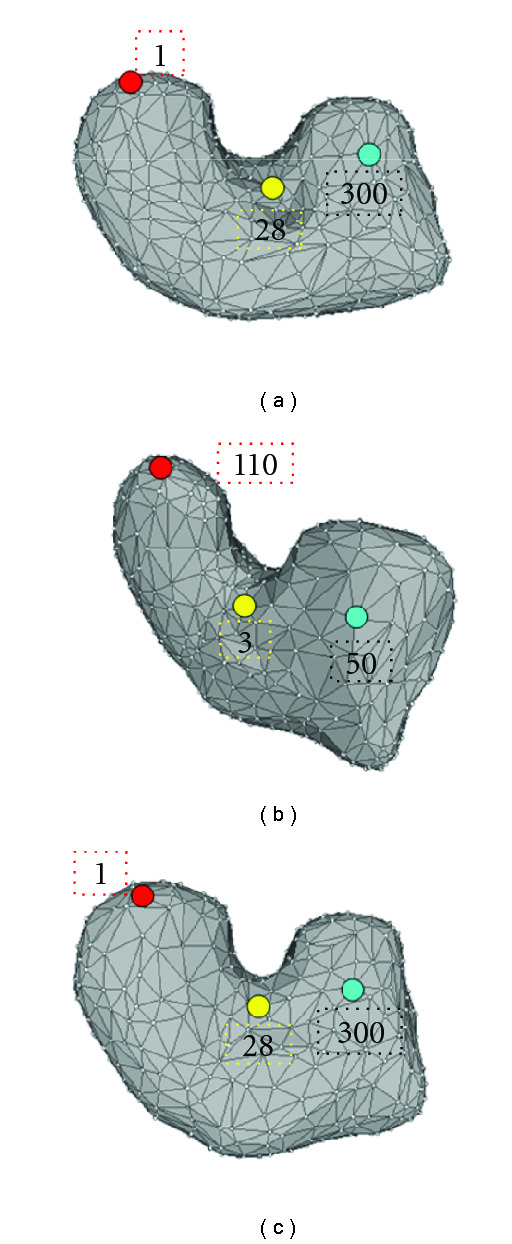
Surface correspondence: (a) vertex index on deformation 1, (b) vertex index on deformation 2 before correspondance, (c) corresponding vertex index on deformation 2.

Similar to other surface modeling methods [25, 27, 28], the proposed approach requires point-wise correspondence across difference surfaces besides rigid registration [34]. Specifically, this point-to-point alignment is established, such that an identical spherical parameterization can be applied in the SHD procedure.  Figure 3 illustrates the goal of surface correspondence. Same colored vertices on deformation 1 and 2 indicate a matched pair. After established correspondence of the point pairs over the two deformations, vertices on deformation 2 can be numbered in the same order as deformation 1.

Different correspondence methods have been proposed, such as minimum description length [35], SH-coefficient alignment [36], and so forth. We applied the SH based method [36] in this paper as well as ray-casting method for simple surfaces. The former SH-based method is based on the underlying fact that two points with the same parameter pair when mapped to a sphere are considered to be a corresponding pair. Therefore, it fixes parameterization of the template and rotate the other to optimize the surface correspondence by minimizing the root mean squared distance of the two SH coefficient vectors. The latter ray-casting method is introduced in the following section.

#### 2.5.1. Point Correspondence with Ray Casting

**Figure 4:**
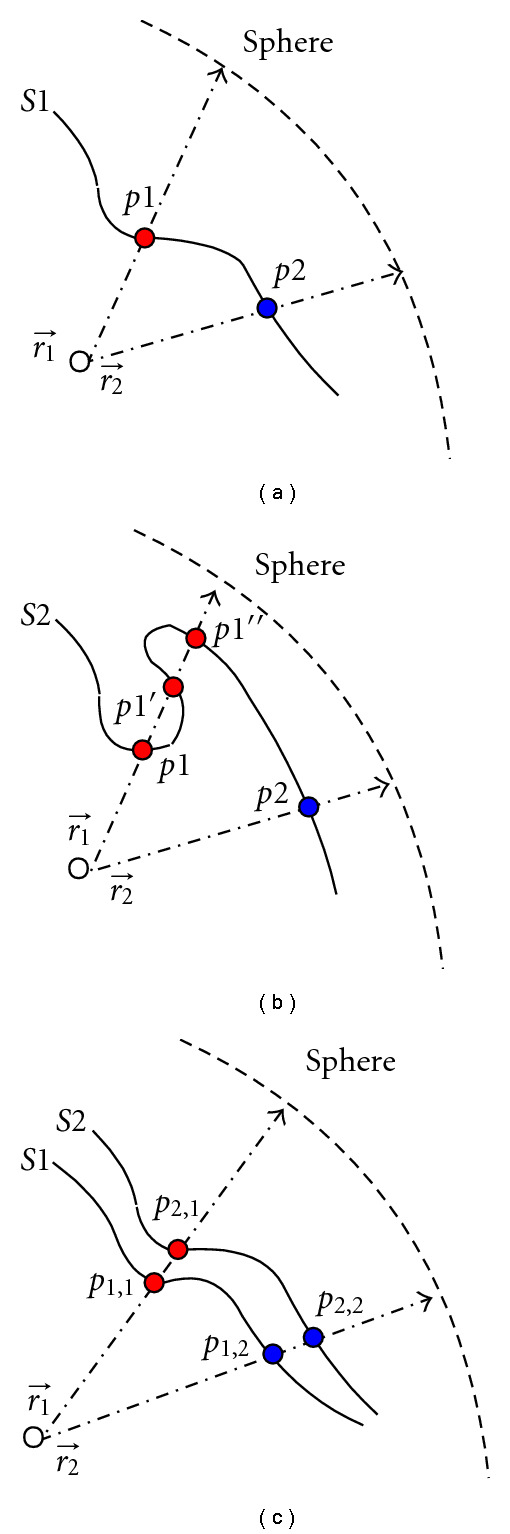
Ray casting sampling: (a) unique ray-surface intersection, (b) multiple ray-surface intersections, and (c) ray casting on two deformed surfaces.

For surfaces, if unique intersection exists between a ray starting from its object center and the surface, a ray-casting method can be applied to obtain sample pairs across all the deformations. For illustration, Figures 4(a) and 4(b) depict two different cases of ray-surface intersection in the simplified 2D space. In Figure 4(a), there is only one intersected point (*p*1 for ray $\vec{r_{1}}$, and *p*2 for ray $\vec{r_{2}}$) between each ray and the deformed surface *S*1. By contrast, Figure 4(b) gives an example when multiple intersections (*p*1, *p*1′, *p*1′′ for ray $\vec{r_{1}}$ and *p*2 for ray $\vec{r_{2}}$) are involved between rays and the surface *S*2. 

If the condition of single ray-surface intersection applies, deformations can be resampled through the following steps to achieve point correspondence.

- Construct an icosahedron of *W* vertices with radius large enough to embrace the largest deformation volume among those under consideration; larger *W* results in denser samples to maintain the local details but incurs more computational cost.
- Align the center of the 3D surfaces to the origin of icosahedron such that rays casting from the origin can intersect with the surface.
- For each ray segment originated from the center to a vertex on the icosahedron, find the triangle on the surface mesh that intersects with the segment and use that intersected point as new surface sample.

**Figure 5:**
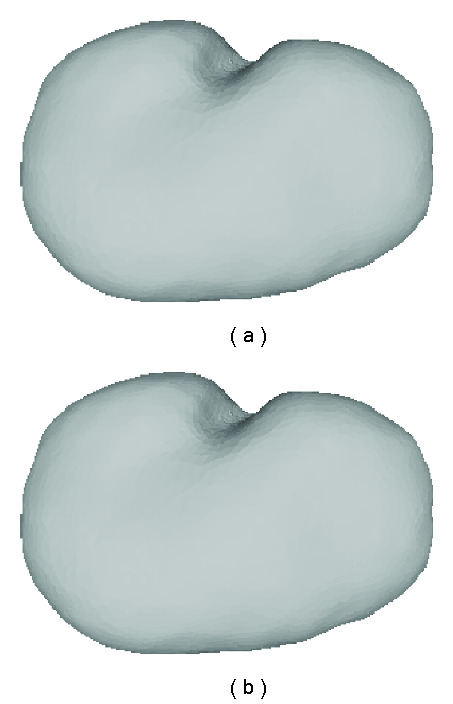
Resample with ray casting (a) original surface of kidney, (b) resampled surface of kidney.

Figure 4(c) illustrates the desired sample pairs as (*p*_1,1_, *p*_2,1_) and (*p*_1,2_, *p*_2,2_) over two deformations *S*1 and *S*2. This resampling process also establishes a one-to-one map between a point on the object and a point on the sphere (icosahedron), which naturally meets the purpose of spherical parameterization. As a result, point-wise correspondence can be achieved across all the resampled surfaces, and a uniform spherical harmonic matrix **Y** can be applied. As an example, Figures 5(a) and 5(b) compare an original kidney surface with the corresponding resampled surface. We can see that ray-casting procedure well maintains the shape.

## 3. Experiments

 Three types of experiments are conducted to demonstrate the feasibility of the proposed SRDS algorithm. The computer-generated FEM data is first used to demonstrate that the SRDS approach matches the accuracy of complex mathematical modeling techniques, then an ex vivo experiment is conducted using 3D MRI scans of porcine kidneys for evaluation in practical settings, and finally in vivo experiment is carried over dynamic cardiac MRI scans for evaluation in real patients.

### 3.1. Experiment with FEM Data

Three representative organs are employed in this FEM experiment: 3D cortical mesh as an example of complicated shapes, gallbladder as an instance with geometrically simple shape, and bladder consisting of both interior and exterior walls.

#### 3.1.1. Computer Model Setup

**Figure 6:**
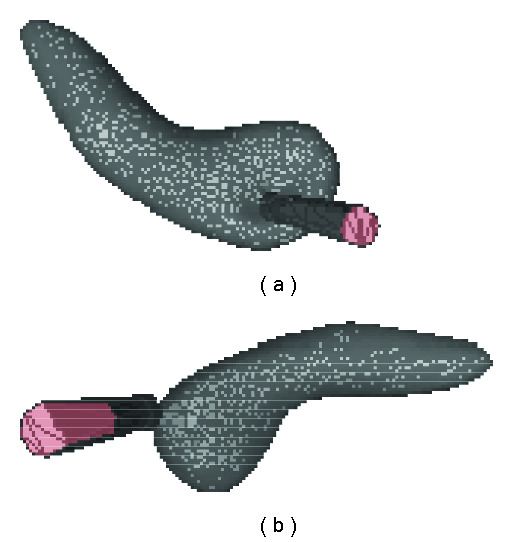
Gallbladder deformation generated by surgical simulator: (a) and (b) are distortions caused when the endoscope pokes and grasps the gallbladder.

The initial 3D models of different organs are fed into a FEM-based surgical simulation tool to generate deformation data for testing. For instance, Figure 6 demonstrates two examples of shape distortions due to the endoscope poking and grasping one side of the gallbladder.

Table 1 lists the FEM experimental setup of the three organs including number of vertices *N*, SH level *L*, number of deformations for training *K*, and testing *M*. “GBL” stands for gallbladder in all the tables. The maximum SH level used for brain model is chosen according to [9], and the levels for gallbladder and bladder are determined when the SHD representation error is below 0.1% (EOF). The complex brain structure requires more vertices and higher SH level for surface representation to achieve sufficient accuracy. To evaluate the representation precision qualitatively, an evaluation parameter EOF is defined as the normalized Euclidean distance between the original surface and the reconstructed surface

(19) $$\begin{array}{c} \text{EOF}=\frac{{||{\hat{x}}_{k}-x_{k}||}_{2}}{{||x_{k}||}_{2}}. \end{array}$$

All surfaces are centered to the origin of the coordinate system so that EOF will not be heavily affected by the denominator.

#### 3.1.2. Results

With the FEM data, the proposed SRDS algorithm is evaluated from three perspectives: (1) subspace dimensionality, (2) sparsity and accuracy of representations, and (3) the effect of subspace pursuit threshold *ɛ* and coefficient truncation threshold *δ* on the performance. The sparsity is defined as the *l*_0_ norm of the coefficient vector ${\tilde{\vec{c}}}_{m}$.

**A. Training Results**

During training stage, we set *ɛ* = 0.005 for subspace detection, *η* = 0.01 for clustering, *E*_max_ = 50 as the maximum iteration times, and *δ* = 0.005 for coefficient truncation. Subspaces on *X*, *Y*, and *Z* axis are identified separately.  Table 2 shows that the subspace number *J* and dimensions of resulting dictionary (dim (*G*) = *I*) are markedly small relative to *N* or *L*^2^ in all three tests. We notice that the subspace dimensions of brain are relatively smaller than the other two. This is because of smaller training data size and minor extent of deformation considered in the brain experiment, which results in smaller dictionary size to capture the deformation features.

**B. Sparsity and Accuracy Evaluation**

Sparsity is examined in terms of (*μ*/*σ*), where *μ* is the average *l*_0_ norm of the coefficient vector ${\tilde{\vec{c}}}_{k}$ (training) or ${\tilde{\vec{c}}}_{m}$ (testing) and *σ* is the corresponding standard deviation. To verify that whether our method achieves equivalent sparsity and precision when applying the structure of the dictionary, we also test the case without relying on any structure learned from training set, during which sparse representation of each deformation in the testing set is repursued from the training set using OSP approach. In the following tables, we use “OSP” to refer to the results obtained using such a repursuing process.  Table 3 summarizes the sparsity of the SRDS representation of three organs for both training and testing set. It illustrates that the number of atoms needed for representing the complex deformations is much smaller than the dimension of spherical harmonic vectors ((*L*+1)^2^), and particularly the sparsity and accuracy via SRDS is very close to that from OSP repursuit for the testing deformations, which indicates the good generalization of the structured dictionary.

**Figure 7:**
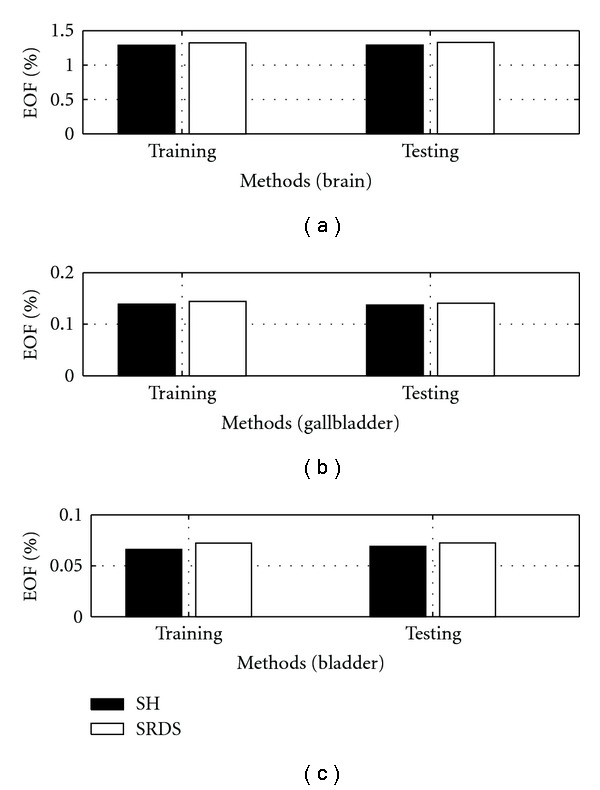
EOF of brain, gallbladder, and bladder reconstruction with SHD and SRDS methods. The left pair is for training set; the right pair is for testing set.

The reconstruction error in terms of EOF is further compared with that from standard SHD method, as shown in Figure 7. In general, the accuracy of SRDS is equivalent to that of SHD method. Specifically, it shows that the SRDS method achieves average EOF of 1.32% (brain) and 0.14% (gallbladder) versus 1.29% (brain) and 0.13% (gallbladder) with SHD method. For bladder model with deformations on multiple layers, the overall representation error with SRDS is 0.07%, very close to 0.06% with SHD. Figures 11 and 12 show typical reconstructed deformations of the testing data for the three organs with SHD and SRDS methods. Figures 12(e) and 12(f) demonstrate the interior and exterior representation of the bladder at a same time instance. From those results, we can see that the SRDS algorithm achieves the accuracy equivalent to complex mathematical modeling techniques while significantly lowers the representation dimensionality.

**C. Effect of *ɛ***

**Figure 8:**
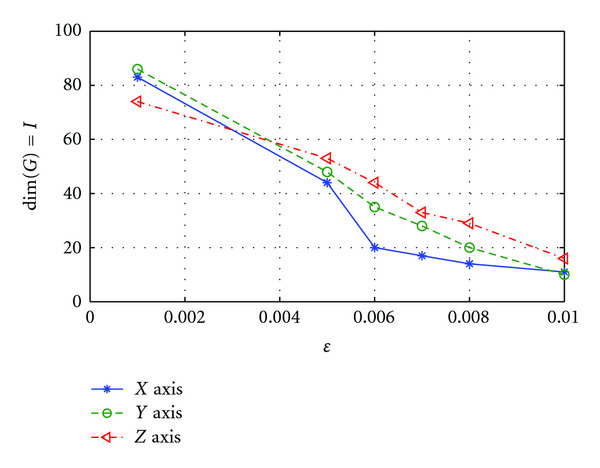
Dimension *I* of trained dictionary *G* decreases on all three axis as *ɛ* increases.

**Figure 9:**
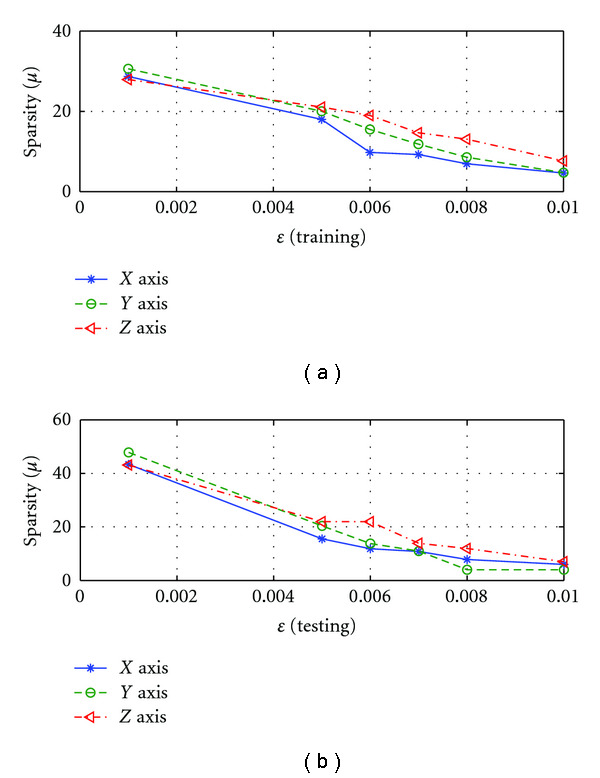
Sparsity (*μ*) of surface representation changes with different *ɛ* on three axis: (a) is for training set; (b) is for testing set.

**Figure 10:**
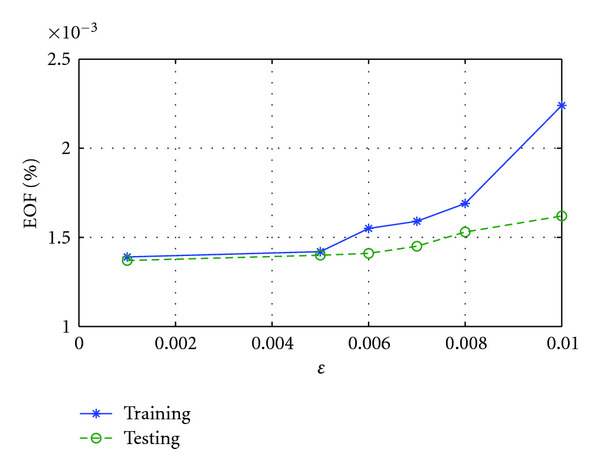
EOF of gallbladder reconstruction increases as *ɛ* gets larger for both training and testing sets.

**Figure 11:**
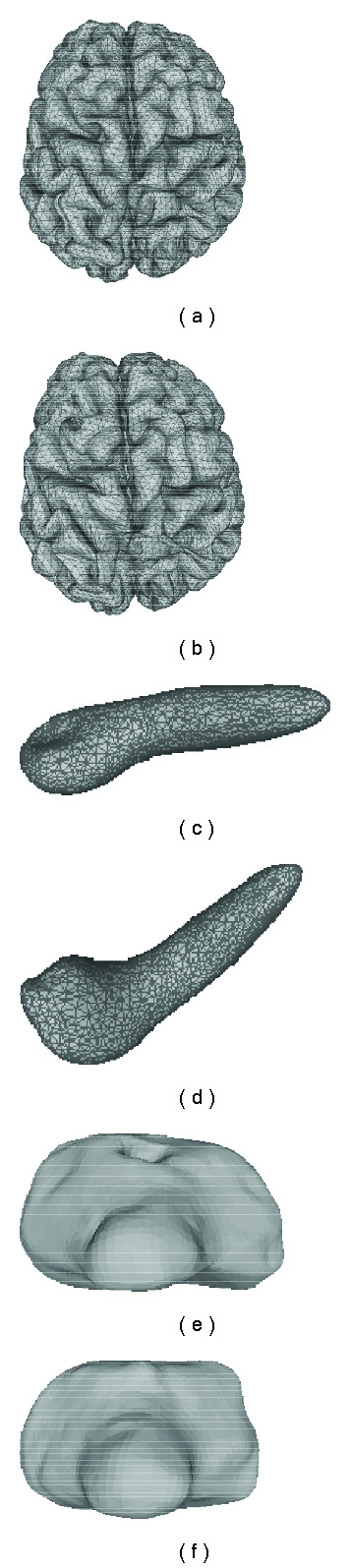
Reconstructed brain deformations using SHD approach: (a) is the initial brain shape and the circle marks one typical area under deformation, (b)–(e) are the reconstructed brain deformations.

**Figure 12:**
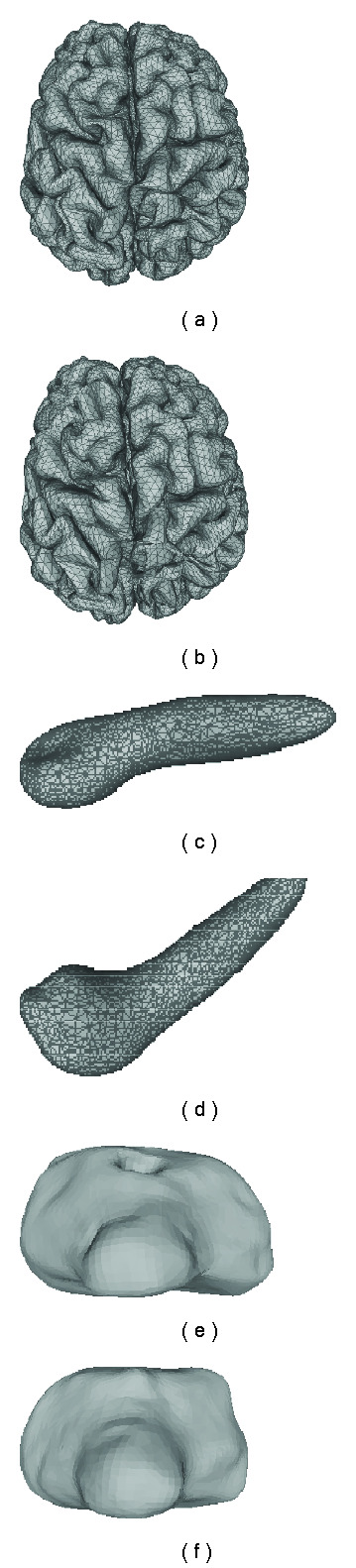
Reconstructed brain deformations using SRDS approach: (a) is the initial brain shape, (b)–(e) are the reconstructed brain deformations.

The performance of SRDS algorithm is examined as the subspace pursuit threshold *ɛ* varies. Specifically, we study the effect of *ɛ* on the dimensionality (*I*) of the structured dictionary *G*, sparsity and accuracy of the surface representation.  Figure 8 shows how the subspace dimensions on three axis change during the training stage as *ɛ* increases from 0.001 to 0.01.  Figure 9 displays the influence of *ɛ* on the average sparsity *μ* of the surface representation results for both training and testing data sets. In general, smaller *ɛ* leads to larger subspace size and less description sparsity, since lower *ɛ* usually leads to more recruited atoms to meet the desired representation accuracy. Therefore, there is a tradeoff between representation accuracy and desired sparsity.  Figure 10 reveals the representation EOF as a function of *ɛ*. Not surprisingly, the reconstruction error is increased as *ɛ* becomes larger. An empirical point can be chosen according to the training curve when space dimension *I* expands significantly but only trivial EOF improvement is gained, that is, *ɛ* = 0.005 is a preferred value in this test according to Figure 10.

**D. Effect of *δ***

**Figure 13:**
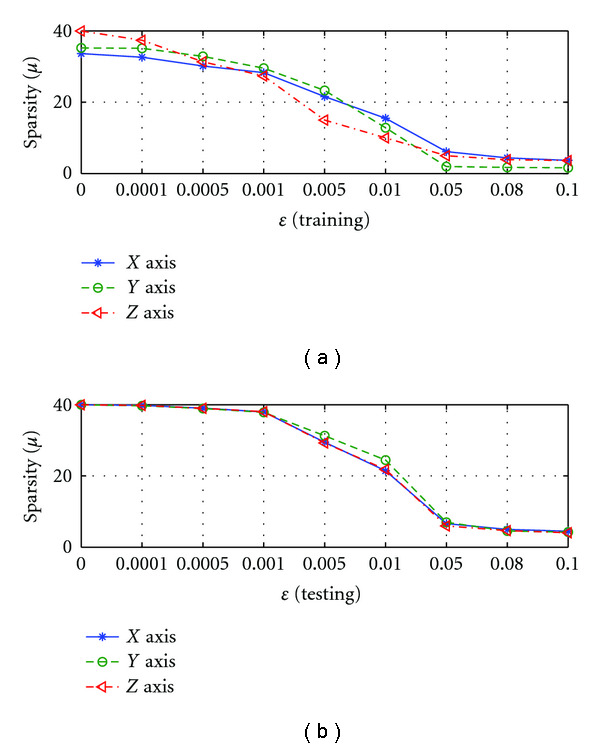
Sparsity (*μ*) of surface representation changes with different *δ* on three axis: (a) is for training set; (b) is for testing set.

**Figure 14:**
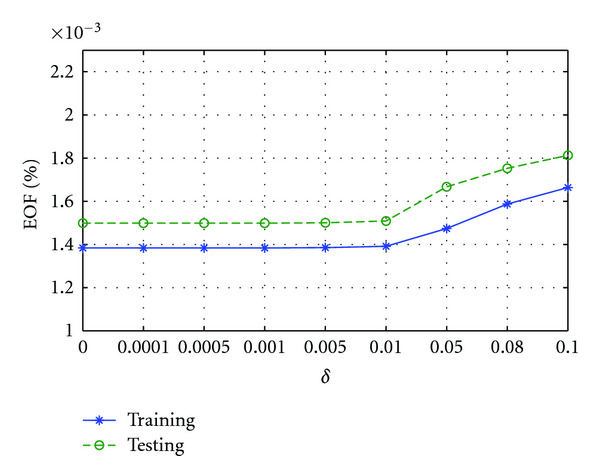
EOF of gallbladder reconstruction increases as *δ* gets larger for both training and testing sets.

The influence of coefficient truncation threshold *δ* on the performance of SRDS algorithm is also tested while *δ* is varied among [00.0001 0.0005 0.001 0.005 0.01 0.05 0.08 0.1].  Figure 13 shows the effect of *δ* on the average sparsity *μ* of the surface representation results. We can see that, as the truncation threshold *δ* enlarges, the sparsity of the representation is increased for both training and testing data sets at the price of decreased representation error as shown in Figure 14, so there is tradeoff between sparsity and accuracy. Empirically, one can choose the *δ* value when the representation precision remarkably deteriorates while the sparsity is still increasing. Therefore, according to Figures 13 and 14, an appropriate value for *δ* is between 0.005 and 0.01.

### 3.2. Ex vivo Experiment Using MRI

**Figure 15:**
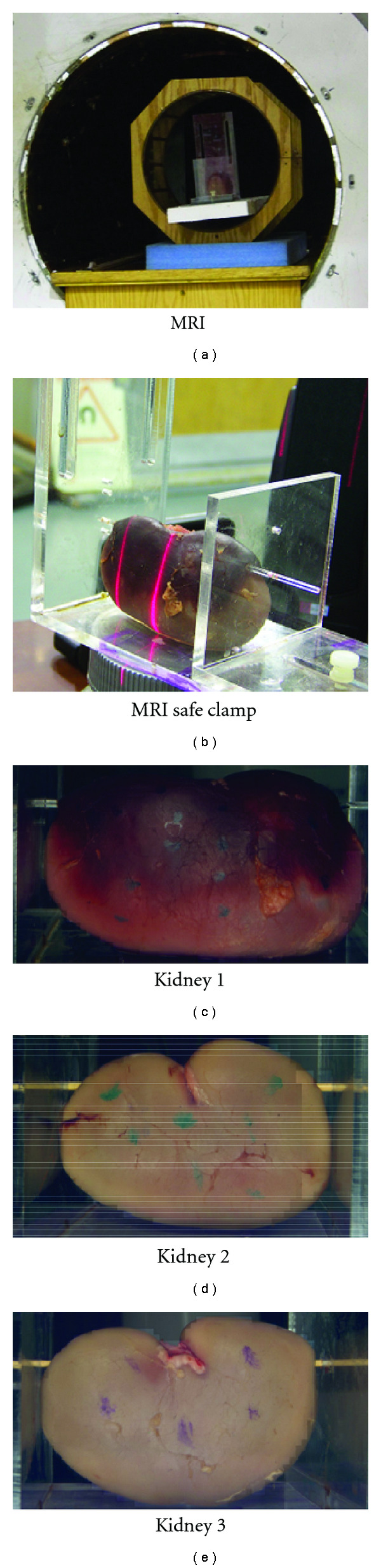
Images of three porcine kidneys for experiment.

To evaluate the proposed algorithm in real applications, an ex vivo experiment using three porcine kidneys were conducted at the Center for Interdisciplinary Applications in Magnetic Resonance (CIA-MR) of University of Minnesota. Deformations imposed to each kidney were controlled and maintained still during imaging by a customized nonmagnetic mechanical device as shown in Figure 15. Each deformed kidney shape was scanned in 3D MRI mode with spatial resolution of 1.2 mm to generate both training set and testing set. The SH degree *L* of the organ representation is set to be 20, and each 3D kidney mesh after surface correspondence has *N* = 4002 vertices. Different from computer-generated deformations where surface correspondence is intrinsically established, the shapes from MRI scans are rendered independently, so the method described in Section 2.5 is applied to achieve point-wise correspondence.

Both intramodel and intermodel experiments are conducted. The former uses training and testing deformations from the same kidney; the later utilizes two out of the three kidneys for training and the third one for testing in a cross-evaluation fashion. Besides sparsity and EOF, Hausdorff distance between the represented shape and corresponding MRI surface is also examined as a physical measurement of error. The Hausdorff distance between surface *x* and *x*′ is defined as

(20) $$\begin{array}{c} d\left(x,x^{\prime }\right)=\underset{p\in x}{\max\;}\;\;d\left(p,x^{\prime }\right), \end{array}$$

where *d*(*p*, *x*′) is defined as the distance between a point *p* on surface *x* and the closest point on surface *x*′, that is,

(21) $$\begin{array}{c} d\left(p,x^{\prime }\right)=\underset{p^{\prime }\in x^{\prime }}{\min\;}\;{||p,p^{\prime }||}_{2} \end{array}$$

with ||·||_2_ denoting the Euclidean norm.

#### 3.2.1. Intramodel Test

In the intramodel experiment, 31 deformations of the same kidney were generated and scanned by the MRI machine, among which 20 frames were randomly selected as training set, and the other 11 were applied for testing the generalization of the learned subspaces.

Table 4 shows the trained subspace dimensions (*J* as number of subspace, *I* as dictionary size of **G**), the sparsity of the descriptors in each axis for both training set and testing set, and the corresponding errors in terms of EOF and Hausdorff distance. Similar to the FEM experiment, the sparsity is also evaluated with OSP repursuit process in the testing set for comparison. The table shows that the sparsity and the accuracy achieved with SRDS is very close to that from OSP repursuing process. However, the SRDS method features delay-free surface representation by applying the structure in the identified dictionary. Further results about computational efficiency are shown in Section 3.4. One may notice that the size of training data in the MRI experiment is smaller than that in FEM test due to the less availability of 3D MRI images. As a rule of thumb, larger training set carries richer deformation information and thus leads to better generalization of the dictionary. However, given the size of training data and extent of deformation involved in the ex vivo experiment, high representation precision is still achieved. 

**Figure 16:**
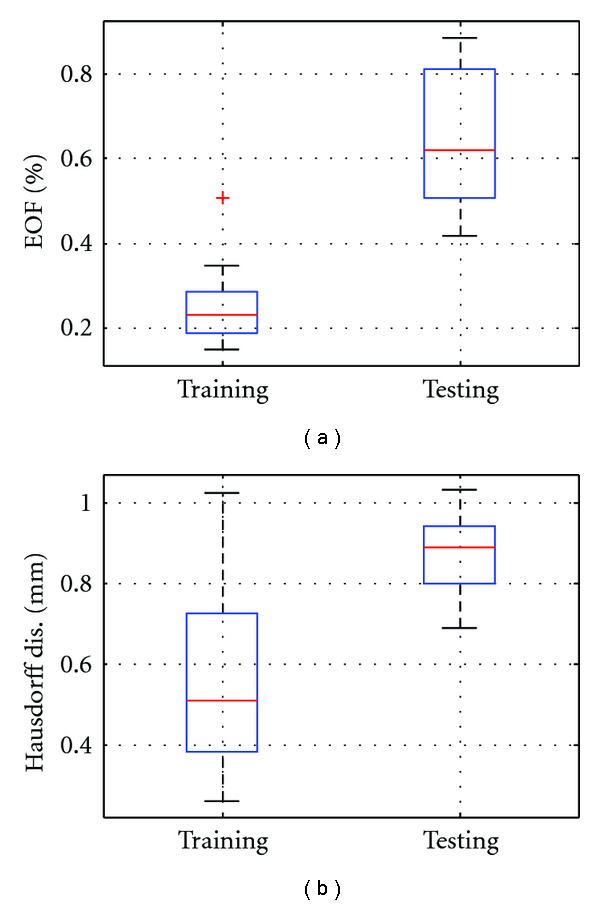
Boxplots of representation error in intramodel experiment: (a) EOF of training and testing set, (b) Hausdorff distance of training and testing sets.

Figure 16 illustrates the accuracy of the surface representation in the intramodel test. The average EOF in Figure 16(a) for training set is 0.24% and 0.64% for testing set, with maximum rate less than 1%. Further, error as Hausdorff distance (shown in Figure 16(b)) is 0.55 ± 0.23 mm with 95th percentile error of 0.86 mm for the training set, and 0.87 ± 0.10 mm with 95th percentile error of 0.96 mm for the testing set. This intramodel experiment demonstrated that the SRDS algorithm identifies subspaces generalizable enough to accurately represent deformations beyond the training set for the same object.

**Figure 17:**
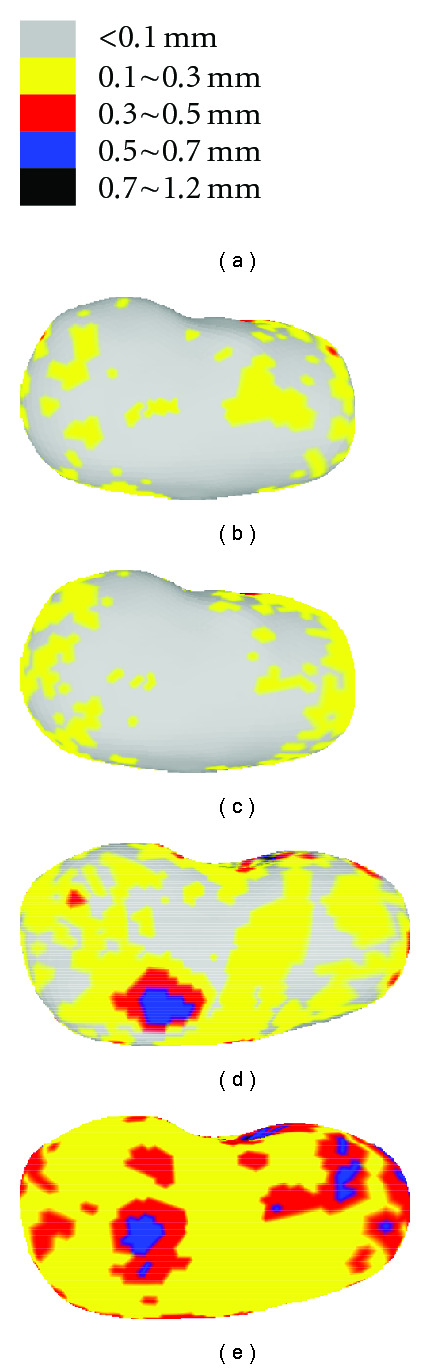
Representation error in intramodel experiment: (a) color-coded scales, (b) example representation error in training set, (c) 90% representation error in training set, (d) example of representation error in testing set, and (e) 90% representation error of testing set.

Figure 17 visualizes the color-coded error distribution at all vertices on the represented surface with SRDS relative to the actual MRI scans.  Figure 17(a) illustrates the error range for different colors. Figures 17(b) and 17(d) show the error distribution for a typical reconstruction in the training and testing set, respectively. Figures 17(c) and 17(e) show maximum 90% level reconstruction errors, that is, 90% of all deformations in the training or testing set have representation point errors less than the values shown in the figures. Consistent with the EOF and Hausdorff distance results, the color diffusion in Figure 17 indicates that the precision in the testing group is relatively lower than that in the training group. However, among all the pixel-wise errors shown in Figure 17(e), less than 3% of all the surface points have error distance larger than 0.5 mm.

#### 3.2.2. Intermodel Test

Three intermodel experiments are performed to further validate the proposed SRDS method applied to organs from different subjects. In the following context, “Ex1” stands for the experiment training on Kidney 2 and 3 plus one initial shape of Kidney 1 while testing on deformations of Kidney 1, and the like for “Ex2” and “Ex3”. In each experiment, both sparsity and accuracy are examined.

The number of subspaces (*J*) and dimensions (*I*) of the identified dictionary are listed in Table 5. The training results vary among the three experiments but all features low subspace dimensions.  Table 6 shows the sparsity of the intermodel experiments using the SRDS algorithm, and the error level is evaluated in terms of EOF and Hausdorff distance. Each testing deformation is also sparsely retrained using OSP for comparison. We can see that the sparsity and representation error resulting from SRDS method is very close to that using OSP.

**Figure 18:**
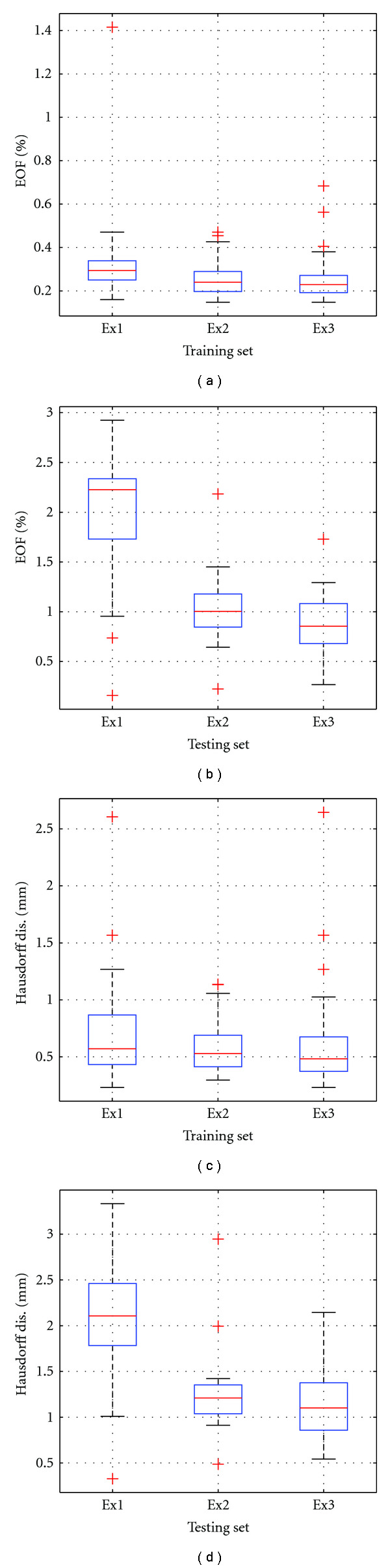
Boxplots of representation error in intermodel experiments: (a) EOF of training set, (b) EOF of testing set, (c) Hausdorff distance of training set, (d) Hausdorff distance of testing set.

Figure 18 shows the representation accuracy using SRDS algorithm in training and testing sets for the three tests. In general, the error in testing set is larger than that in the training set. Particularly, as for EOF evaluation, “Ex1” leads to the largest EOF error relative to “Ex2” and “Ex3”, but the average error rate is still as low as 0.3% for training set and 2.0% for testing set.  Table 7 lists the specific Hausdorff measurements corresponding to boxplots in Figures 18(c) and 18(d), including minimum, 95th percentile and mean. We can see that the 95th percentile Hausdorff distance across all experiments is below 3 mm, and the mean is belong 2.1 mm. Comparing those error levels with the intramodel test, one can see that the homology existing among the training and testing deformations contributes to better dictionary generalization and, thus, leads to higher representation accuracy.

**Figure 19:**
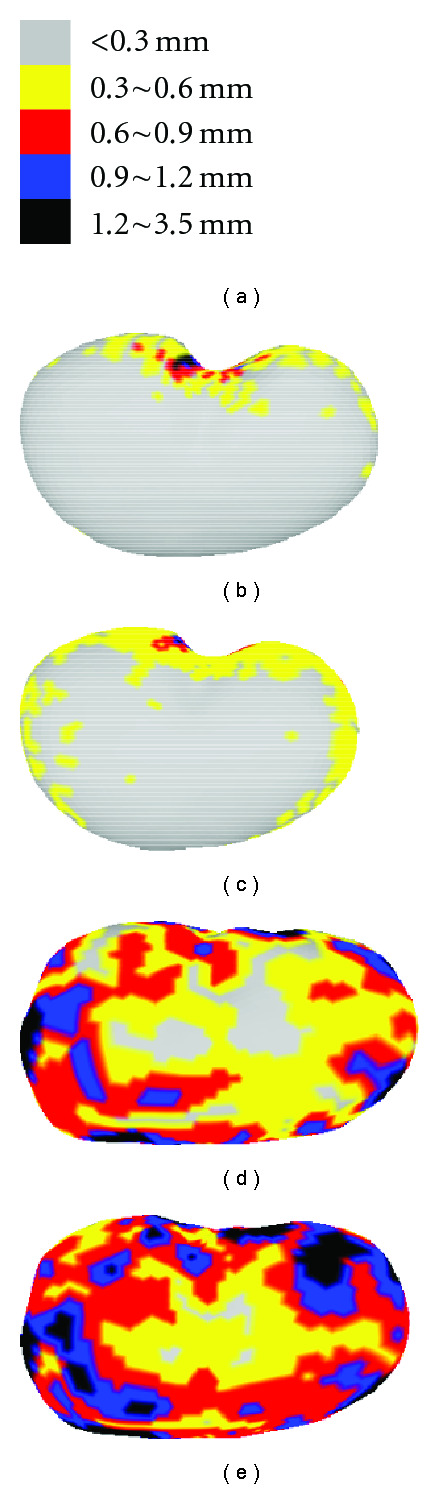
Representation error in intermodel Ex1: (a) color-coded scales, (b) example of color-coded point error in training set, (c) 90% color-coded point error in training set, (d) example of color-coded point error in testing set, and (e) 90% color-coded point error of testing set.

**Figure 20:**
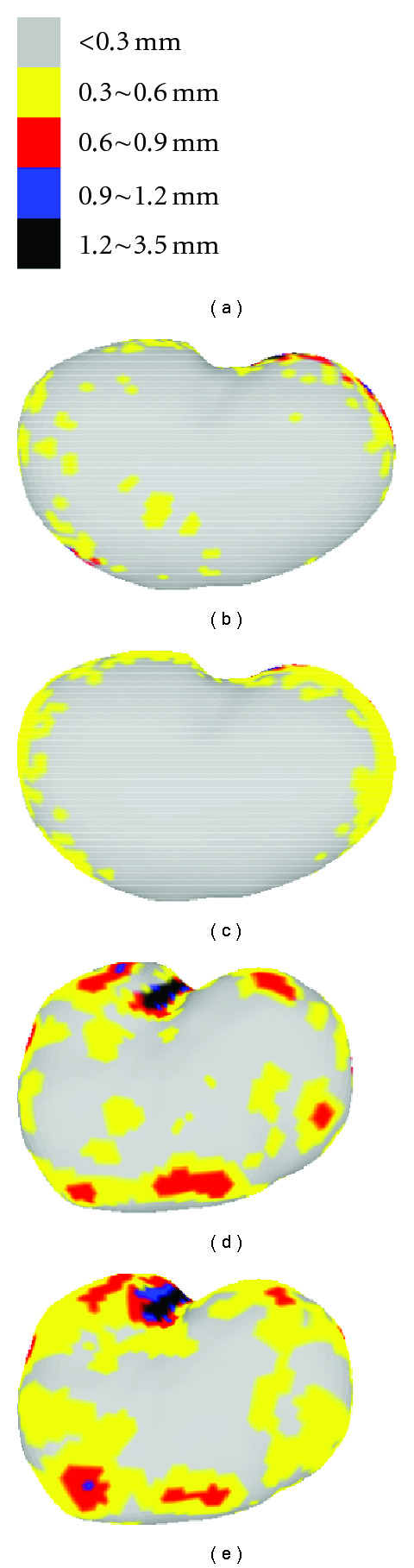
Representation error in intermodel Ex2: (a) color-coded scales, (b) example of color-coded point error in training set, (c) 90% color-coded point error in training set, (d) example of color-coded point error in testing set, and (e) 90% color-coded point error of testing set.

**Figure 21:**
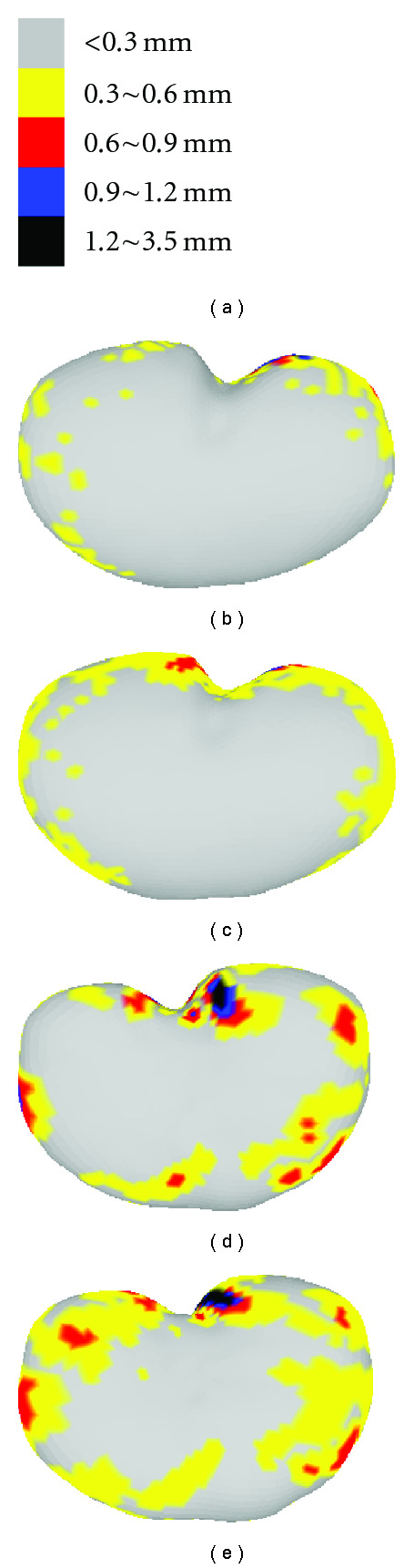
Representation error in intermodel Ex3: (a) color-coded scales, (b) example of color-coded point error in training set, (c) 90% color-coded point error in training set, (d) example of color-coded point error in testing set, and (e) 90% color-coded point error of testing set.

Figures 19, 20 and 21 show the color-coded error fields of a typical representation and at the maximum 90% level for the three intermodel experiments. In either training set or testing set, it is observed that large errors are mostly distributed around the edge area where local details are rich. Consistent with the previous EOF and Hausdorff distance measurements, the color diffusion in Figures 19–21 indicates that errors in testing set is larger than that in training set and “Ex1” generates relatively larger error comparing to “Ex2” or “Ex3.”

### 3.3. In Vivo Experiment Using MRI

The proposed approach is also tested over the in vivo cardiac MR images [37], consisting of automatically segmented images from volumetric MRI scans of a diastole-systole-diastole cycle. For each patient, there are around 22 phases in a cardiac cycle. Surface correspondence of LV shapes within and across patients are accomplished using the approach described in Section 2.5. Since the generated surfaces from automatic segmentation software are quite rough, we use the spherical harmonic representation as a filter to smooth out those surface noises and then apply the smoothed surfaces as training and testing data. Therefore, the demonstrated error in this section is relative to the SHD surfaces, not to the original raw surfaces. The iter-patient results are reported as follows.

Similar to the ex vivo test, we use the segmented left ventricles (LV) of 2 different patients plus an initial LV surface for the third patient as training data, and the remaining LV shapes in a beating cycle of the third patient are used to test the generalization of the identified subspaces. The formulated tests are noted as “Ex1,” “Ex2,” and “Ex3.” Table 8 lists the sparsity test results of the three cross validations for both training and testing sets. We can see that the sparsity in the training set is close to that in the testing set, but the former achieves much higher accuracy. This is because that the identified subspaces generalize perfectly for those elected atoms among the training set after spherical harmonic smoothing. Consistent with the previous experiments, the representation of testing surfaces using SRDS is also compared with that using repursuing OSP. According to the results, SRDS achieves performance slightly worse than but close to that of OSP. However, as demonstrated in Section 3.4, without relying on the structured dictionary learned from the training population, OSP is a computational expensive task, since for each testing surface, it requires to research for atoms from the training set to achieve sparse representation.

**Figure 22:**
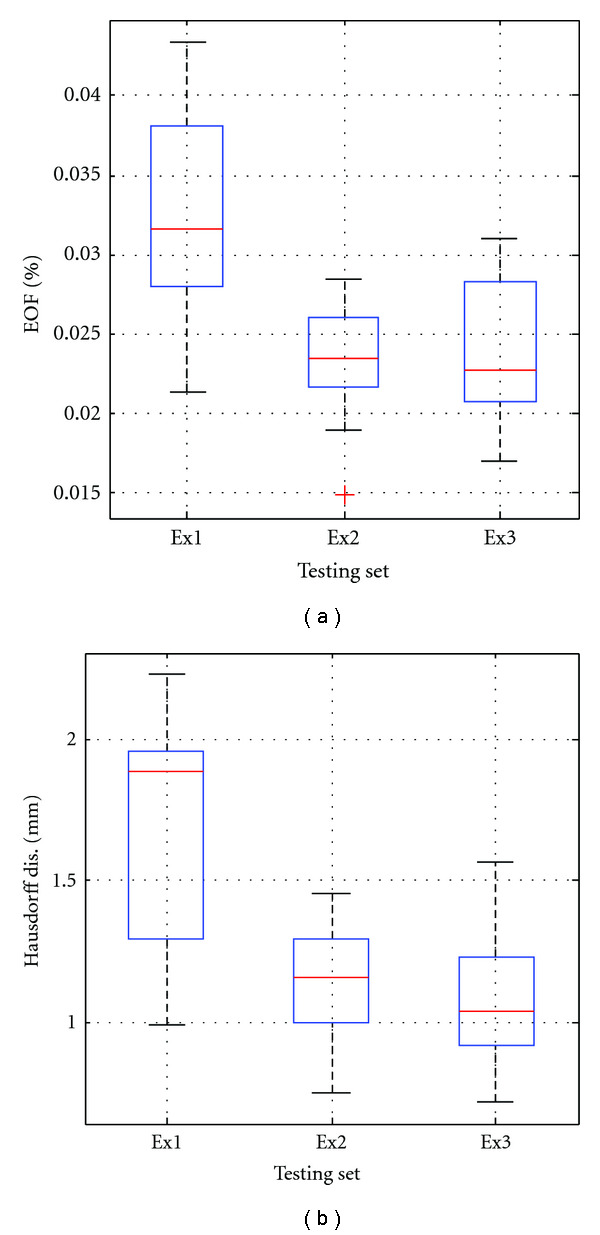
Boxplots of representation error in LV interpatient experiments: (a) EOF, (b) Hausdorff distance.

**Figure 23:**
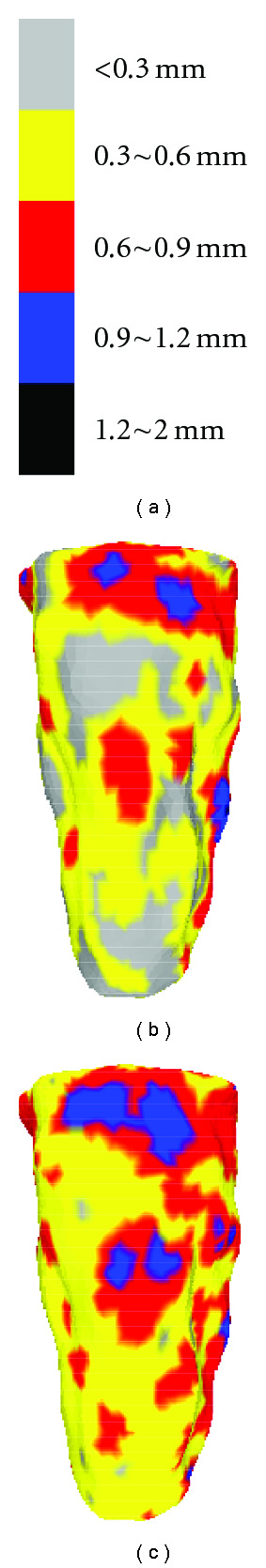
Representation error in in vivo experiment: (a) color-coded scales, (b) example representation error in testing set, (c) 90% representation error of testing set.

Figure 22 provides boxplots for the representation accuracy of the testing set in terms of EOF and Hausdorff distance.  Table 9 provides the minimum, 95th percentile, and mean Hausdorff measurements corresponding to Figure 22(b). In coincidence, “Ex1” leads to slightly larger errors than the other two tests, with average EOF of 3.2% (“Ex1”), and mean Hausdorff distance of 1.67 ± 0.39 mm. The 95th percentile Hausdorff distance across all experiments is below 2.2 mm. Figures 23(b) and 23(c) show the color-coded error field of a typical representation and at the maximum 90% level for the testing set in one interpatient experiment (c). As indicated by the color distribution, majority of the point errors are below 0.9 mm. Particularly, in the 90th percentile evaluation in Figure 23, only 3% of all the point-wise errors are above 0.9 mm.

### 3.4. Efficiency

To examine the efficiency of the proposed SRDS method quantitatively, the computational time to represent each surface in the testing set using SRDS method is compared with that resulting from OSP repursuing approach for the above five organs. The results are summarized in Table 10, including training set size *K*, maximum SH level *L*, average time (in seconds) required with SRDS (*t*_1_) and OSP (*t*_2_), respectively, and the ratio between the two. As shown in Table 10, the time consumption for seeking sparse representation of the testing surfaces using the SRDS is at least 10 times lower than that using the original OSP method which does not rely on the dictionary structure learned from the training data set. The advantage is more pronounced when the training data size *K* or the SH level *L* is large. For example, in the brain model, the high SH level *L* leads to substantial computational delay during the search for proper atoms for representation, such that the SRDS achieves a speed orders of magnitude faster than the OSP method without training. On the other hand, for the case of gallbladder, the large training size also increases the time used by repursuing OSP, so it runs 65 times slower than SRDS.

To summarize, considering the test results for sparsity, accuracy, and efficiency given in this experiment section, we can see that the proposed SRDS method achieves sparse surface representation with high computational efficiency and accuracy.

## 4. Conclusions and Discussion

This paper introduced a new algorithm for block sparse representation of deformable organ surfaces with high accuracy. The proposed SRDS design first identifies the deformation subspaces from the training data set in the transformed spherical harmonic domain and then represents each deformed surface with a block sparse vector in the structured dictionary. SRDS is generalized to applications involving organs with multiple surface layers, such as bladder. The algorithm has been validated with FEM data and real 3D MRI scans under both ex vivo and in vivo conditions. The FEM test results demonstrate that SRDS achieves accuracy matching that of complex mathematical modeling techniques. Further, the maximum representation error in ex vivo experiment is below 1 mm for intramodel test and below 3 mm for intermodel test. For the in vivo experiment, the SRDS achieves an accuracy of better than 2.5 mm.

SRDS algorithm has already been used in tracking organ deformations in minimum invasive surgery [2–4]. The structure introduced in the dictionary enables efficient surface recovery from limited samples. In addition, the merits of block sparse surface representation presented here can be applied to various medical organ modeling, shape classification, and similarity retrieval where reduced parameter dimension can potentially speed up the implementations.

## Figures and Tables

**Table 1:** FEM Model setup.

	Vertices *N*	SH level *L*	Training *K*	Testing *M*
Brain	40962	80	35	35
GBL	3038	25	250	114
Bladder	*N*1 = 4434,	30	74	46
	*N*2 = 4274			

**Table 2:** Dimension of dictionary (*J*/*I*).

	Brain	GBL	Bladder
Subspace on *X* (*J*/*I*)	2/13	2/39	1/37
Subspace on *Y* (*J*/*I*)	2/6	2/48	1/30
Subspace on *Z* (*J*/*I*)	1/3	1/42	1/34

**Table 3:** Sparsity (*μ*/*σ*) and accuracy evaluation.

	*X* (*μ*/*σ*)	*Y* (*μ*/*σ*)	*Z* (*μ*/*σ*)	EOF (%)
Brain train (SRDS)	3.1/0.2	3.4/0.9	3.0/0.2	1.31
Brain test (SRDS)	3.5/0.5	3.9/0.4	3.0/0.0	1.32
Brain test (OSP)	4.1/0.6	4.5/0.5	3.9/0.8	1.30

GBL train (SRDS)	25.8/12.2	4.8/1.8	32.3/7.3	0.13
GBL test (SRDS)	33.2/1.0	44.1/1.1	40.8/1.3	0.15
GBL test (OSP)	44.4/4.0	48.3/2.3	43.9/4.5	0.13

Bladder train (SRDS)	24.1/3.7	20.8/2.9	24.8/4.0	0.076
Bladder test (SRDS)	24.6/2.7	21.0/2.5	25.6/3.2	0.073
Bladder test (OSP)	22.5/4.3	16.3/6.5	23.0/5.2	0.070

**Table 4:** Subspace dimension and sparsity (*μ*/*σ*) for intramodel experiment.

	*X* (*μ*/*σ*)	*Y* (*μ*/*σ*)	*Z* (*μ*/*σ*)	EOF (%)	Haus (mm)
*J*/*I*	1/17	2/31	2/31		
Train (SRDS)	16.9/0.4	15.5/1.5	15.8/1.5	0.24	0.55
Test (SRDS)	17.0/0.0	16.9/0.3	16.7/0.9	0.64	0.87
Test (OSP)	16.6/1.5	16.3/0.9	17.1/0.8	0.60	0.85

**Table 5:** Subspace dimension (*J*/*I*) in intermodel experiments.

	*X* axis	*Y* axis	*Z* axis
Ex1 (*J*/*I*)	1/36	1/37	1/37
Ex2 (*J*/*I*)	2/87	2/86	2/87
Ex3 (*J*/*I*)	3/79	2/88	3/83

**Table 6:** Sparsity (*μ*/*σ*) and accuracy evaluation for intermodel experiment with kidneys.

	*X* (*μ*/*σ*)	*Y* (*μ*/*σ*)	*Z* (*μ*/*σ*)	EOF (%)	Haus (mm)
Ex1 train (SRDS)	34.7/5.5	35.6/5.5	36.4/1.6	0.32	0.69
Ex1 test (SRDS)	35.7/1.3	36.7/0.9	36.7/1.8	2.01	2.08
Ex1 test (OSP)	36.7/2.4	36.0/2.4	35.9/2.6	1.94	1.94

Ex2 train (SRDS)	42.8/4.5	42.8/2.2	43.5/3.7	0.26	0.59
Ex2 test (SRDS)	47.0/3.1	44.5/1.8	46.3/2.4	1.02	1.27
Ex2 test (OSP)	44.0/2.2	41.9/12.1	45.9/2.0	0.95	1.22

Ex3 train (SRDS)	37.1/9.1	43.3/2.2	40.8/1.3	0.25	0.59
Ex3 test (SRDS)	41.4/6.5	45.1/1.7	40.4/1.3	0.90	1.18
Ex3 test (OSP)	41.2/8.4	42.7/9.7	39.3/10.6	0.83	1.16

**Table 7:** Hausdorff distance for intermodel experiment with kidneys.

	Min (mm)	95th (mm)	Mean (mm)
Ex1 train	0.23	1.14	0.69 ± 0.42
Ex1 test	0.33	2.81	2.08 ± 0.61

Ex2 train	0.30	1.03	0.59 ± 0.23
Ex2 test	0.49	1.99	1.27 ± 0.49

Ex3 train	0.23	1.03	0.59 ± 0.39
Ex3 test	0.54	1.74	1.18 ± 0.44

**Table 8:** Sparsity (*μ*/*σ*) along *X*, *Y*, *Z* axis and accuracy of in vivo LV tests.

	*X* (*μ*/*σ*)	*Y* (*μ*/*σ*)	*Z* (*μ*/*σ*)	EOF (%)	Haus (mm)
Ex1 train (SRDS)	37.0/0.0	35.4/0.5	31.7/9.0	0.13	0.08
Ex1 test (SRDS)	37.0/0.0	36.0/0.0	34.6/1.5	3.21	1.07
Ex1 test (OSP)	33.3/10.5	36.8/2.6	33.8/9.2	2.92	0.98

Ex2 train (SRDS)	35.4/7.4	32.8/14.1	32.4/3.3	0.17	0.10
Ex2 test (SRDS)	38.0/0.0	37.0/0.0	34.0/0.0	2.31	1.15
Ex2 test (OSP)	32.1/6.3	24.0/10.5	32.3/6.5	1.93	1.07

Ex3 train (SRDS)	36.7/3.2	38.8/1.5	38.4/0.5	0.12	0.07
Ex3 test (SRDS)	39.6/1.2	40.0/0.0	38.8/0.4	2.33	1.09
Ex3 test (OSP)	32.7/11.5	33.4/11.3	30.1/12.2	1.98	0.99

**Table 9:** Hausdorff distance for in vivo experiment with LV.

	Min (mm)	95th (mm)	Mean (mm)
Ex1 train	0	0.50	0.08 ± 0.18
Ex1 test	0.99	2.16	1.67 ± 0.39

Ex2 train	0	0.63	0.10 ± 0.24
Ex2 test	0.75	1.37	1.14 ± 0.18

Ex3 train	0	0.60	0.07 ± 0.21
Ex3 test	0.72	1.46	1.09 ± 0.24

**Table 10:** Computational time of SRDS and OSP.

	Training *K*	SH level *L*	SRDS (sec) *t*_1_	OSP (sec) *t*_2_	*t*_2_/*t*_1_
Brain	35	80	0.3	51.2	170.7
GBL	350	25	0.6	39.1	65.2
Bladder	74	30	2.5	57.2	22.9
Kidney	52	20	0.8	9.2	11.5
LV	51	25	0.5	6.9	13.8
